# Pelota: A double-edged sword in virus infection

**DOI:** 10.1371/journal.ppat.1013328

**Published:** 2025-07-10

**Authors:** Xue Li, Xueping Zhou, Fangfang Li

**Affiliations:** 1 State Key Laboratory for Biology of Plant Diseases and Insect Pests, Institute of Plant Protection, Chinese Academy of Agricultural Sciences, Beijing, China; 2 State Key Laboratory of Rice Biology, Institute of Biotechnology, Zhejiang University, Hangzhou, Zhejiang, China; Virginia Polytechnic Institute and State University, UNITED STATES OF AMERICA

## Abstract

Pelota, a conserved ribosome rescue factor involved in mRNA surveillance, has emerged as a pivotal player in host–virus arms race. Beyond its canonical role in maintaining translational fidelity via No-Go Decay and Non-Stop Decay pathways, Pelota exhibits a dual function during viral infection-serving either as a restriction factor or as a susceptibility element depending on the virus species and their hosts. In DNA virus infections, notably with geminiviruses, a natural mutation in Pelota confers recessive resistance in tomato and pepper probably by impairing viral protein translation, offering valuable insights for resistance breeding. Conversely, in RNA virus infections, Pelota usually restricts viral propagation through RNA quality control, yet can also promote viral replication by facilitating ribosome recycling and translation. This paradox reflects a fine-tuned balance in host–virus adaption and co-evolution. Additionally, *pelota* mutations can modulate immune signaling pathways, with some alleles triggering enhanced resistance or autoimmunity phenotypes in plants. Meanwhile, viruses have evolved counterdefense strategies, including targeted degradation or SUMOylation interference, to subvert the Pelota’s function. Together, these findings position Pelota as a double-edged sword in viral infection, highlighting its potential as a novel target for antiviral strategies through precise genetic manipulation.

## Introduction

Viral infection heavily relies on the host translation machinery for viral protein synthesis, making host translation regulatory factors crucial players in virus–host interactions [1,2]. Among these, components of mRNA surveillance and ribosome recycling pathways not only maintain cellular homeostasis but also contribute to antiviral defense by selectively targeting viral RNAs for degradation or impeding their translation. Additionally, RNA quality control (RQC) system is an interface where host defense and viral exploitation intersect. In eukaryotes, Pelota forms a functional complex with the GTPase Hbs1 responsible for ribosome rescue and mRNA surveillance, thus to assure the quality and fidelity of mRNA molecules. Understanding how these systems, particularly the core effector proteins of RQC, Pelota-Hbs1 complex, function during viral infection provides new perspectives in the evolutionary arms race between host and virus interactions.

## Pelota and its role in mRNA surveillance

**Fig 1:**
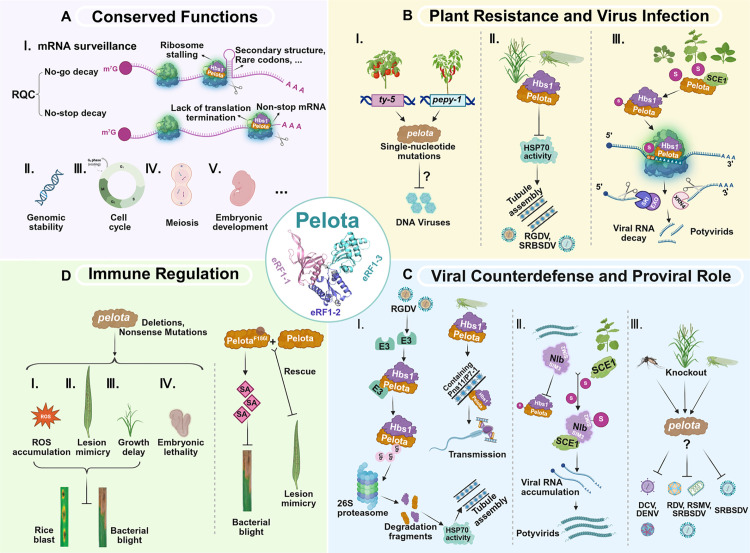
The dual roles of Pelota in mRNA surveillance, viral infection, and plant immunity. **(A)** Conserved functions of Pelota. Pelota forms a heterodimer with Hbs1 to resolve stalled ribosomes in No-Go Decay (NGD) and Non-Stop Decay (NSD) pathways. The complex triggers endonucleolytic cleavage of aberrant mRNAs, followed by their degradation. Pelota also regulates genomic stability, cell cycle, meiosis, and embryonic development in animals. **(B)** Pelota’s role in DNA/RNA virus infection and resistance. (Ⅰ) Recessive resistance to geminiviruses (e.g., TYLCV in tomato *ty-5*; PepYLCIV in pepper *pepy-1* via impaired ribosome recycling. (Ⅱ, Ⅲ) Antiviral actions against RNA viruses (e.g., OsPelota inhibits Hsp70-dependent tubule assembly; viral RNA degradation via SUMOylation-dependent RQC). **(C)** Viral counterdefense and Pelota’s proviral roles. (Ⅰ, Ⅱ) Viral evasion strategies (e.g., E3 ligase degrades Pelota-Hbs1, RGDV Pns11 binds to Pelota enabling tubule assembly; NIb suppresses Pelota SUMOylation). (Ⅲ) Proviral functions in viral translation (e.g., DCV capsid synthesis; DENV, RDV, RSMV, SRBSDV replication). **(D)** Pelota mutations in immune regulation. Deletions or nonsense mutations cause severe immune activation conferring resistance; OsPelota^F186I^ activate SA pathway. TYLCV: tomato yellow leaf curl virus; PepYLCIV: pepper yellow leaf curl Indonesia virus; RGDV: rice gall dwarf virus; SRBSDV: Southern rice black-streaked dwarf virus; DCV: *Drosophila* C virus; DENV: dengue virus; RDV: rice dwarf virus, RSMV: rice stripe mosaic virus. Created in BioRender. Li, X. (2025) https://BioRender.com/vgdppvs.

Pelota (Dom34 in *Saccharomyces cerevisiae*) is an evolutionarily conserved mRNA surveillance factor involved in ribosome recycling to ensure proper ribosome turnover after translation termination. In plants, animals, and *S. cerevisiae*, Pelota forms a heterodimeric complex with the GTPase Hbs1 (Hsp70 subfamily B suppressor1), functioning as a key component of the closely related No-Go Decay (NGD) and Non-Stop Decay (NSD) pathways, two important RNA surveillance systems in eukaryotes (Fig 1A-Ⅰ) [3–5]. This complex is responsible for recognizing and resolving stalled ribosomes, thereby maintaining translational fidelity and preventing the accumulation of aberrant RNA and protein products. When abnormal ribosome behavior is perceived, the surveillance complex triggers endonucleolytic cleavage of the faulty mRNA. Following this cleavage, the 3′ RNA fragment is rapidly degraded by the 5′–3′ exonuclease XRN4, whereas the 5′ fragment is processed by a 3′–5′ exonuclease complex in a SKI2-dependent manner (Fig 1A-Ⅰ, B-Ⅲ) [6]. Moreover, studies in animals have shown that Pelota also plays a crucial role in regulating the cell cycle, embryonic development, meiosis, growth, and maintaining genomic stability (Fig 1A-Ⅱ–Ⅴ) [7–9].

Structurally, Pelota is a conserved protein that shares significant similarity with eukaryotic release factor 1 (eRF1). The overall architecture of three eRF1 domains resembles a tRNA molecule. Correspondingly, Pelota eRF1-like domain can be divided into three conserved segments: the N-terminal domain (NTD; eRF1-1), the central M domain (eRF1-2), and the C-terminal domain (eRF1-3) (Fig 1, the central circle) [10,11,14].The eRF1-like domain shares structural similarity with the translation termination factor eRF1, enabling Pelota to mimic eRF1 interaction with stalled ribosomes [3,12]. This structural feature is essential for its role in recognizing aberrant translation complexes. The M domain is responsible for binding Hbs1, and the NTD domain may contribute to substrate specificity or interaction with other factors in the surveillance pathway [4]. These domains are evolutionarily conserved across *S. cerevisiae*, animals, and plants, highlighting the universal importance of Pelota in translational quality control [3]. Of note, subtle mutations, especially in the N-terminal region, can affect Pelota’s ribosome binding and decay, potentially altering antiviral defense [10,13,14].

In *Arabidopsis*, AtPelota1 participates in both NGD and NSD pathways, and Pelota is emerging as more than a housekeeping factor. As shown in several recent studies, Pelota can be co-opted by viruses or mobilized by the host as part of the immune machinery, depending on the specific virus–host interaction (Fig 1B, C). This functional versatility makes Pelota an intriguing target for further investigation in antiviral strategies.

## Pelota is a susceptibility factor during the infection of plant DNA viruses

Although Pelota has a predominant role in RNA surveillance, it also contributes to the infection of plant DNA viruses, particularly geminiviruses, through mechanisms that appear to involve translational control rather than RQC monitoring alone. The tomato yellow leaf curl virus (TYLCV) is a monopartite begomovirus (family *Geminiviridae*) with single-stranded circular DNA genome, and the causal agent of the destructive tomato leaf curl disease [15–17]. Previous genetic studies have identified naturally occurring *pelota* mutations that confer strong recessive resistance to begomoviruses. A key example is the *ty-5* locus in tomato (*Solanum lycopersicum*), which encodes SlPelota. In the TYLCV-resistant line TY172 and AVTO1227, a T-to-G substitution at position 47 in the first exon results in a V16G amino acid change in Pelota, conferring robust resistance to TYLCV and other begomoviruses (Fig 1B-Ⅰ ) [13,18,19]. Overexpression of the susceptible allele in TY172 restores susceptibility, whereas overexpressing the resistant allele in susceptible lines has no effect, which indicates that Pelota^V16G^ acts through a recessive resistance mechanism [13].

Notably, unlike *Ty-1*-mediated resistance, which can be overcome by co-infection of TYLCV with a betasatellite [20,21], *ty-5*-mediated resistance remains effective even against begomovirus/betasatellite complexes. The *ty-5* gene confers resistance not only to the two representative begomoviruses-tomato yellow leaf curl China virus (TYLCCNV)/tomato yellow leaf curl China betasatellite (TYLCCNB) and tomato leaf curl Yunnan virus (TbLCYnV)), but also to a curtovirus beet curly top virus (BCTV). Moreover, *Nicotiana benthamiana* was transitioned from being susceptible to geminivirus infection to becoming resistant upon the knockdown of *PELOTA* expression. Therefore, *ty-5* with the V16G amino acid mutation in Pelota confers broad-spectrum resistance to various geminiviruses [22].

A similar mechanism has been reported in pepper (*Capsicum annuum*), where the *pepy-1* gene, also encoding Pelota, confers resistance to pepper yellow leaf curl Indonesia virus (PepYLCIV) and pepper yellow leaf curl Aceh virus (PepYLCAV), two geminiviruses. Whole-genome re-sequencing identified an A-to-G mutation at the 9th intron splice site of *CaPelota*, resulting in intron retention and the addition of 28 amino acids without frameshift. This variant restricts viral DNA replication by altering the Pelota’s function without fully abolishing it (Fig 1B-Ⅰ) [23].

Although the precise mechanism by which Pelota restricts geminivirus infection remains elusive, current evidence suggests that *pelota* mutations impair ribosome recycling and reduce translation efficiency, particularly in infected cells, thus limiting virus proliferation [13,24]. It is also plausible that these mutations disrupt host–virus translational coupling, or affect Pelota’s interaction with the viral genome or replication-associated proteins-avenues that remain underexplored. The identification of Pelota variants conferring broad-spectrum resistance in tomato and pepper opens up promising possibilities for resistance breeding. However, given Pelota’s central role in essential cellular processes, including mRNA surveillance and ribosome homeostasis, any modification must avoid fitness penalties. Engineering resistance through Pelota requires a fine-tuned balance between antiviral efficacy and host viability.

## The dual role of Pelota in regulating the infection of RNA viruses

Unlike its proviral role in DNA virus infections, Pelota plays a far more nuanced role in RNA virus infection, where it can act as either an antiviral factor or a proviral host dependency.

### Pelota restricts RNA viruses by exploiting the RNA surveillance system

The Pelota/Dom34-mediated RQC targets virus RNA for degradation via the NGD pathway [26,28]. During the Gag translation of human immunodeficiency virus, host factor RVB2 binds to the nascent Matrix domain and the 5′ UTR of the translating mRNA, causing ribosome stalling and recruitment of Pelota to initiate mRNA degradation [25]. Depurinated brome mosaic virus (BMV) RNA3 associates with polysomes in vivo, where ribosomes stall at the depurination site and are subsequently recognized by the Dom34/Hbs1 complex of the NGD pathway, leading to translation-dependent accelerated degradation of the viral RNA [26]. In leafhoppers, the Pelota-Hbs1 complex on sperm targets rice gall dwarf virus (RGDV)-containing tubules via Pns11-Pelota interaction and inhibits Hsp70-dependent tubule assembly (Fig 1B-Ⅱ) [14]. The Pelota-Hbs1 complex defends against rice viruses in both rice and insect by directly interacting with the Southern rice black-streaked dwarf virus (SRBSDV)-encoded tubular protein P7-1. Notably, OsPelota overexpression in transgenic rice confers broad resistance to rice dwarf virus (RDV) and rice stripe mosaic virus (RSMV) infections with an unknown mechanism (Fig 1B-Ⅱ) [27].

The evolutionarily conserved Pelota-Hbs1 complex serves as a negative regulator of plant virus infection in the *Potyviridae* family (potyvirids), the largest group of plant-infecting RNA viruses responsible for over half of global crop losses due to viral diseases [28]. Pelota, a key component of RQC, recognizes the conserved functional G_1–2_A_6–7_ motif within the P3 cistron, allowing it to specifically target potyvirids and act as a viral restriction factor by facilitating the degradation of the genomic RNA. Additionally, Pelota interacts with the SUMO E2-conjugating enzyme SCE1 and undergoes SUMOylation in planta. Disrupting Pelota SUMOylation impairs its ability to recruit Hbs1, thereby inhibiting viral RNA degradation (Fig 1B-Ⅲ, 1C-Ⅱ) [28].

Collectively, these findings highlight the evolutionarily conserved role of Pelota as a key component of host RQC, functioning across species and viral types to restrict RNA viruses through diverse yet mechanistically related pathways. Despite the emerging mechanistic insights, several questions remain unresolved. For instance, whether Pelota targets all RNA viruses with G_1–2_A_6–7_-rich regions with equal efficiency, and how host-specific factors modulate its activity. Additionally, while current studies underscore its antiviral roles in both plants and insect vectors, the extent to which Pelota integrates with other immune signaling pathways remains to be elucidated. Intriguingly, the central role of Pelota in antiviral defense makes it a prime target for viral suppression.

### Viral counterdefense to subvert the Pelota’s function

Viruses, as highly adaptable cellular parasites, have evolved diverse virulence strategies to suppress, manipulate, or evade this defense mechanism. For instance, the RGDV-activated E3 ligase degrades Pelota-Hbs1, while the viral protein Pns11 counteracts this by competing for E3 binding, thereby enabling tubule assembly and facilitating efficient virus transmission (Fig 1C-Ⅰ) [14]. During SRBSDV propagation in both rice and insect vectors, a slight reduction in Pelota levels appears to prevent the excessive inhibition of P7-1 tubule formation, ensuring optimal virus propagation (Fig 1C-Ⅰ) [27]. Moreover, the viral RNA-dependent RNA polymerase NIb acts as a SUMOylation decoy, competitively binding to the host SCE1 and thereby suppressing Pelota SUMOylation, which ultimately inhibits Pelota-mediated RQC (Fig 1C-Ⅱ) [29]. These counter-acting strategies reflect an ongoing evolutionary arms race, in which viruses actively fine-tune rather than entirely abolish the Pelota’s activity. This strategic modulation allows them to escape immune surveillance while maintaining the host functions necessary for viral fitness. Such dynamic regulation underscores the complexity of virus–host interactions, where Pelota typically viewed as an antiviral factor can also be subverted by viruses for their own benefit. Intriguingly, this paradoxical relationship becomes particularly apparent when considering Pelota’s role in promoting viral translation, as discussed in the following section.

### Pelota’s proviral role through promoting viral translation

Despite its well-established antiviral function, Pelota also exhibits a seemingly contradictory role by facilitating viral replication under certain conditions. As a host factor critical for high-efficiency translation, Pelota contributes to the synthesis of viral proteins by maintaining ribosome availability and resolving stalled translation complexes. In *Drosophila*, Pelota deficiency restricts the replication of *Drosophila* C virus (DCV), a positive-sense single-stranded RNA virus, by specifically limiting capsid protein synthesis without significantly affecting the production of other viral or cellular proteins. This restriction likely stems from Pelota’s role in resolving stalled 80S ribosomes and clearing aberrant viral RNA and proteins (Fig 1C-Ⅲ) [30].

A similar phenomenon is observed in *Aedes aegypti*, where *Pelota* expression is upregulated during Dengue virus (DENV) infection, and its knockdown significantly reduces virion production. However, in *Wolbachia*-infected female mosquitoes, Pelota’s expression is downregulated, its subcellular localization is altered, and it may be regulated by aae-miR-2940-5p, potentially contributing to *Wolbachia*-mediated DENV inhibition [31]. Specifically, *SfPelota* knockdown in the planthopper (*Sogatella furcifera*) reduces the transcript and protein accumulation levels of SRBSDV P7-1, thereby inhibiting viral propagation. In rice, knockout of *OsPelota* significantly decreases the accumulation of RDV Pns10 and RSMV N proteins compared to wild-type ZH11 plants, thus impeding viral propagation (Fig 1C-Ⅲ) [27].

Together, these findings reveal a functional duality of Pelota in virus–host interactions. While Pelota-mediated mRNA surveillance plays a defensive role by degrading aberrant viral RNAs, its excessive inhibition paradoxically disrupts ribosome homeostasis, leading to a reduced pool of free ribosomes and consequently impairing viral protein synthesis and replication. This highlights Pelota as both a gatekeeper of RNA quality and an enabler of viral gene expression. Understanding how viruses and hosts modulate the Pelota’s activity may offer novel insights into the fine-tuning of host translation machinery and uncover new targets for antiviral intervention.

## Pelota’s negative regulation role in plant immunity

Given the delicate balance between Pelota’s antiviral and proviral roles, it is not surprising that genetic mutations in *Pelota* can profoundly alter immune responses. This functional plasticity raises a key question: how do natural or engineered mutations shape Pelota’s dual functions in plant immunity? Disrupting this balance through mutation may shift Pelota’s function toward immune activation or repression, depending on the mutation type and biological context. Several studies have identified spontaneous or induced mutations on *pelota* that trigger enhanced immune responses in rice. For instance, the *pelota* spotted-leaf mutant (flanked by two insertion/deletion markers) derived from an EMS-induced rice IR64 mutant bank, exhibits cell death, H_2_O_2_ accumulation, growth arrest, and enhanced broad-spectrum resistance to multiple races of *Xanthomonas oryzae* pv. *Oryzae* (Fig 1D) [32]. Similarly, the *pelota* mutant *lml1-1*, *lml1-2*, and *lml1-3* lines with insertions or deletions in the fifth exon, resulting in amino acid changes or a premature stop codon, also cause cell death and defense activation against both bacterial blight and rice blast (Fig 1D) [11]. In addition, a T-to-A substitution at position 556 in the *OsPelota* coding sequence results in the OsPelota^F186I^ mutation, which confers bacterial blight resistance by activating the salicylic acid pathway (Fig 1D) [33]. Interestingly, *Pelota* deficiency in mice leads to embryonic lethality (Fig 1D) [7], possibly due to dysregulation of immune homeostasis during development. Taken together with the phenotypes observed in plants – such as H₂O₂ accumulation and cell death – we propose that *Pelota* deficiency may trigger uncontrolled immune responses in both animals and plants, highlighting its essential role in maintaining both immune and developmental balance. Furthermore, as previously mentioned, in susceptible tomato cultivars, the *Ty-5* gene encodes the wild-type Pelota. By contrast, resistant lines harbor the *ty-5* allele, which encodes the Pelota^V16G^ variant that confers resistance to TYLCV. Importantly, the *ty-5* allele mediates virus tolerance rather than absolute immunity. These findings suggest that single-nucleotide mutations can fine-tune Pelota’s function to enhance pathogen tolerance, and confer resistance, whereas deletions or nonsense mutations often tend to cause severe immune activation phenotypes such as ROS accumulation, cell death and lesion mimicry.

## Concluding remarks

At the molecular level, mutations in Pelota can influence plant antiviral immunity by disrupting RNA surveillance, altering mRNA degradation efficiency, or rewiring immune signaling pathways. Depending on the nature of the mutation, Pelota can act either as a gain-of-function or loss-of-function factor-serving as a molecular “double-edged sword” in host–virus interaction. This dual role emphasizes the complexity of Pelota’s involvement in both antiviral defense and viral facilitation in the context of different viruses. The intricate relationship between Pelota and plant immunity emphasizes the necessity for further research into how these variations affect the broader antiviral response. Therefore, systematic screening of natural or engineered Pelota variants across crop germplasm could accelerate the identification of antiviral alleles. Coupled with precision genome editing tools, such as CRISPR-Cas systems, these insights may enable the development of disease-resistant cultivars through targeted modulation of the Pelota’s activity. Nevertheless, both overexpression and knockout strategies targeting Pelota entail trade-offs. Overexpression may enhance antiviral defense in certain contexts, but it also raises the risk of Pelota being hijacked by viruses to facilitate their replication. Conversely, *Pelota* knockout or silencing can restrict viral propagation, as shown in insects and rice, but may trigger unintended immune activation or impair normal plant development. To address these challenges, strategies involving context-dependent regulation, including inducible expression systems or tissue-specific editing, should be explored to optimize antiviral outcomes while minimizing adverse effects on plant fitness. However, balancing antiviral efficacy with host fitness is crucial, since Pelota also plays essential roles in cellular processes. In conclusion, the functional plasticity of Pelota, along with its evolutionary conservation, positions it as a critical factor in plant-virus arms race. As research into Pelota’s dual roles continues to unravel, it will undoubtedly provide new avenues for antiviral strategies and contribute to a deeper understanding of the molecular interplay between plants and viruses.

## Abbreviations

**:**
BCTV: beet curly top virus
BMV: brome mosaic virus
DCV: *Drosophila* C virus
DENV: dengue virus
eRF1: eukaryotic release factor 1
NGD: no-go decay
NSD: non-stop decay
PepYLCIV: pepper yellow leaf curl Indonesia virus
PepYLCAV: pepper yellow leaf curl Aceh virus
RGDV: rice gall dwarf virus
RSMV: rice stripe mosaic virus
RQC: RNA quality control
SRBSDV: southern rice black-streaked dwarf virus
TYLCCNV: tomato yellow leaf curl China virus
TYLCCNB: tomato yellow leaf curl China betasatellite
TYLCV: tomato yellow leaf virus
